# Anaplastic thyroid cancer with long-term survival with lenvatinib therapy and preservation of laryngeal function after one-stage reconstruction: A case report

**DOI:** 10.3892/mco.2021.2320

**Published:** 2021-06-11

**Authors:** Akihisa Tanaka, Hirokazu Uemura, Takashi Masui, Shigenori Kanazawa, Yumi Yoshii, Masatoshi Kanno, Gohei Morita, Chiho Obayashi, Toshiaki Yamanaka, Tadashi Kitahara

**Affiliations:** 1 Department of Otolaryngology-Head and Neck Surgery, Nara Medical University, Kashihara, Nara 634-8522, Japan; 2 Department of Otorhinolaryngology-Head and Neck Surgery, Nippon Life Hospital, Osaka-city, Osaka 550-0006, Japan; 3 Department of Cancer Genomics and Medical Oncology, Nara Medical University, Kashihara, Nara 634-8522, Japan; 4 Department of Diagnostic Pathology, Nara Medical University, Kashihara, Nara 634-8522, Japan

**Keywords:** laryngotracheal reconstruction, ATC, lenvatinib, one-stage reconstruction, laryngeal function

## Abstract

Laryngotracheal reconstruction is performed to treat locally advanced thyroid carcinoma invading the larynx and/or trachea. The reconstructive technique varies. The present report describes the case of a 71-year-old female patient who underwent surgery for thyroid carcinoma involving the larynx. Reconstructive surgical techniques were employed to maintain laryngeal structure and function. An anterolateral thigh flap with free rib cartilage grafts was used to compensate for laryngeal defects. Although a temporary tracheal stoma was constructed, it closed spontaneously after decannulation. Therefore, one-stage laryngeal reconstruction was accomplished. Post-operative histopathological examination revealed focal anaplastic changes in the lesion, which mainly consisted of papillary components. Post-operative positron emission tomography/computed tomography indicated early recurrence in the left side of the neck. Therefore, lenvatinib was started as adjuvant therapy. Complete response was observed with lenvatinib therapy. The patient was alive and had good laryngeal function 26 months after the operation. One-stage laryngeal reconstruction can reduce burden and improve quality of life in patients with thyroid carcinoma involving the larynx. Lenvatinib may be useful for treating early recurrence of anaplastic thyroid carcinoma after reconstructive surgery with a free flap.

## Introduction

Locally advanced thyroid carcinoma can involve the larynx (1,2). In such cases, partial laryngotracheal resection is often performed as surgical treatment. Successful reconstruction is required to avoid palliative stenting or permanent tracheostomy. An important aspect of laryngotracheal reconstruction is the establishment of skeletal support in order to resist airway pressure changes (3). Although there are several surgical techniques for performing laryngotracheal reconstruction, surgeons occasionally find it difficult to maintain an appropriate laryngeal and/or tracheal shape.

Anaplastic thyroid carcinoma (ATC) is a rare but highly aggressive malignant tumour that accounts for 1-2% of all thyroid malignancies (4). Although ATC is treated with a multidisciplinary approach that includes surgery, radiotherapy, and chemotherapy, its prognosis is extremely poor. Lenvatinib, a tyrosine kinase inhibitor, has been approved for use in patients with unresectable or metastatic thyroid carcinoma. The use of post-operative lenvatinib therapy has not yet been established, and further studies are required to confirm the efficacy of this therapy.

It is known that ATC may not only originate *de novo* but also arise as a conversion of differentiated thyroid carcinoma (5). Head and neck surgeons may sometimes encounter ATC incidentally when treating patients with locally advanced differentiated thyroid carcinomas (6).

We report a case of incidental ATC that invaded the larynx, with long-term survival with lenvatinib therapy and post-operative preservation of laryngeal function.

## Case report

### 

#### Case presentation

**Figure 1:**
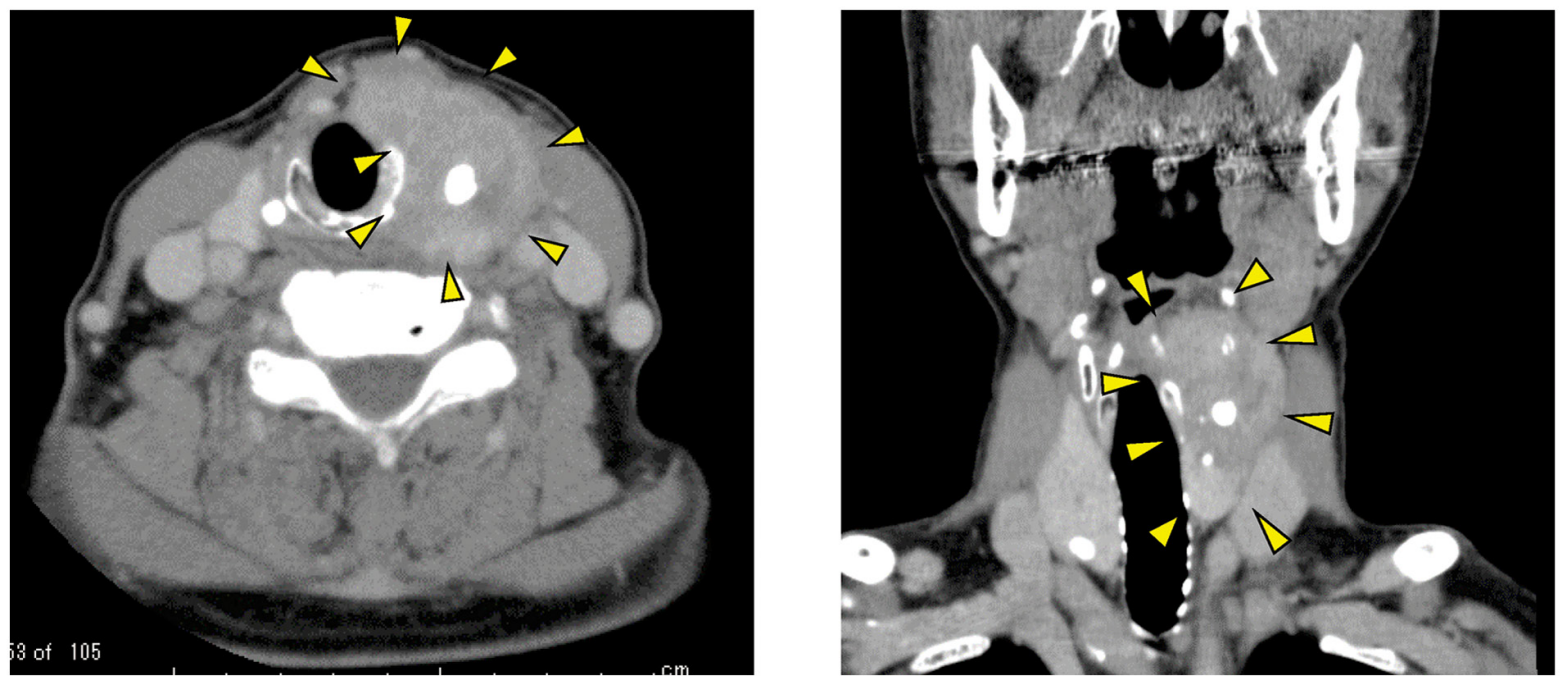
Pre-operative computed tomography images revealed a calcified thyroid tumour (surrounded by arrowheads). The tumour was invasive and extended from the left side of the thyroid cartilage to the upper part of the cricoid cartilage.

A 71-year-old woman visited Nara Medical University Hospital with complaints of hoarseness and a left-sided neck mass (45x30 mm) in October 2018. Left vocal cord paralysis was confirmed endoscopically. The larynx was displaced to the right by a slow-growing left thyroid lobe tumour. We were unable to identify the border between the larynx and the tumour. Computed tomography showed a calcified thyroid tumour extending from the left side of the thyroid cartilage to the upper part of the cricoid cartilage (Fig. 1). Positron emission tomography/computed tomography showed no evidence of distant metastasis. The tumour was histologically diagnosed as papillary thyroid carcinoma (PTC) with no evidence of poorly differentiated carcinoma or ATC. Subsequently, the patient was diagnosed with PTC (cT4aN1bM0, stage III).

#### Surgery

Total thyroidectomy with hemi-laryngectomy and neck dissection was performed. The laryngeal defect was reconstructed using a prefabricated anterolateral thigh (ALT) flap and free rib cartilage grafts in order to maintain laryngeal function.

**Figure 2:**
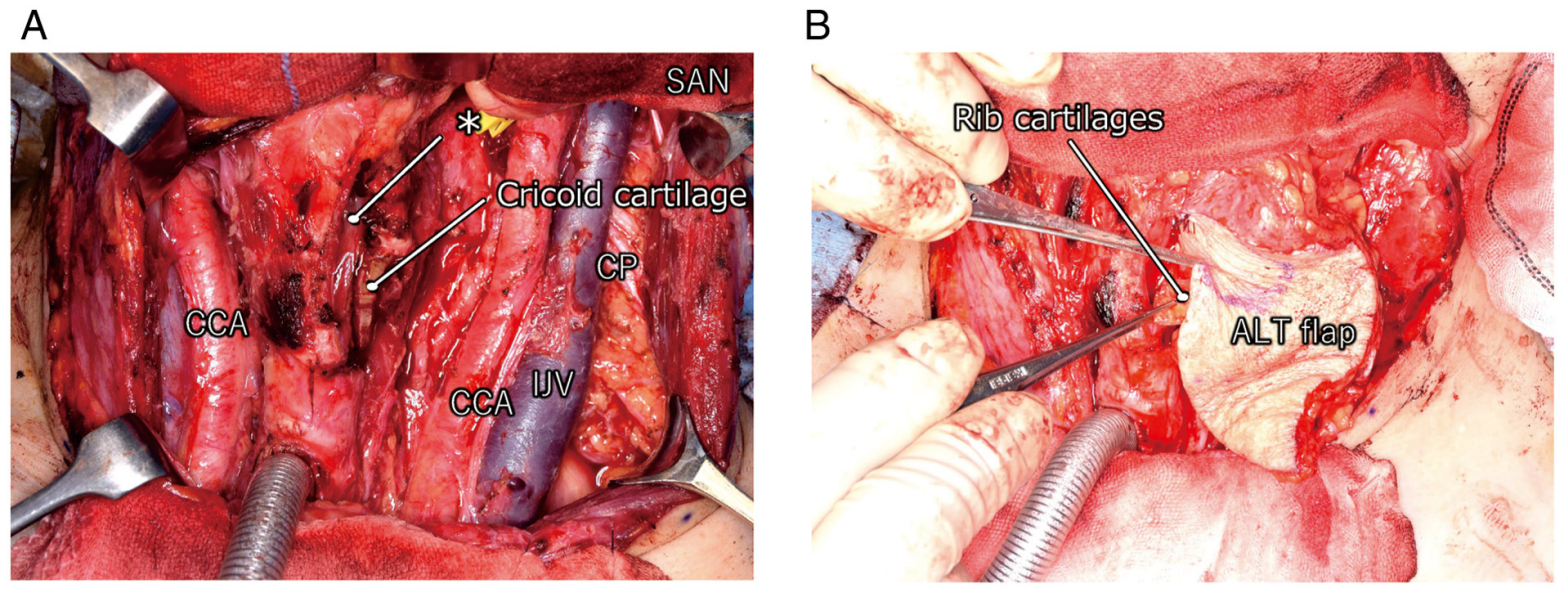
Intraoperative findings. (A) Laryngeal defect is indicated by^*^. (B) Rib cartilage was inserted into a subcutaneous burrow under the ALT flap. ALT, anterolateral thigh; CCA, common carotid artery; CP, cervical plexus; IJV, internal jugular vein; SAN, spinal accessory nerve.

After tumour excision, bilateral paratracheal and left lateral neck dissection was performed. The dissected tissues were gathered toward the thyroid, and the thyroid was dissected from the trachea with relative ease. We resected from the thyroid cartilage to the superior edge of the cricoid cartilage in order to complete en bloc extirpation of the lesion because the tumour involved these structures. We resected approximately 40% of the laryngeal defect, which would have resulted in loss of airway and phonatory function. To compensate the laryngeal defect, grafting using an ALT flap and free rib cartilage was performed as follows: First, the left ALT flap (15x7 cm) was harvested and laid over the surgical defect, and microscopic vascular anastomoses were established. The flap had two perforators based on the left lateral circumflex femoral artery descending branch. The left superior thyroid artery was used as the recipient artery and the branch of the left external jugular vein was used as the recipient vein. Subsequently, small pieces of rib cartilage were harvested from the left side of the chest. The cartilage pieces were placed in the subcutaneous burrow under the ALT flap (Fig. 2). This tissue and the laryngeal remnant constituted the reconstructed larynx that was fully functional.

**Figure 3:**
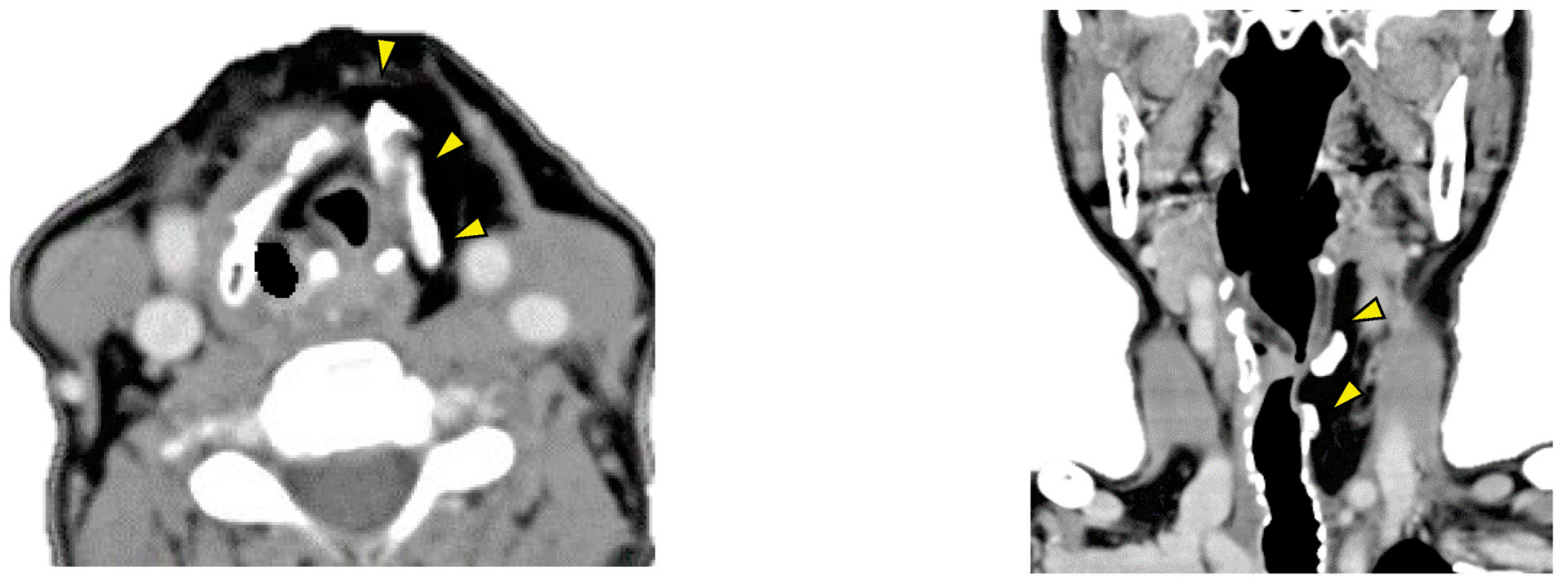
Post-operative computed tomography images show a patent laryngeal lumen. Arrowheads indicate rib cartilages.

On the sixth post-operative day, the patient started oral intake based on videofluorography findings. On the seventh post-operative day, she was able to phonate with a speech cannula. Four months later, the patient was decannulated, and the stoma spontaneously closed. Consequently, one-stage reconstruction was successfully accomplished (Fig. 3).

#### Outcome and follow-up

Post-operative histopathological examination of the tumour revealed that the tumour was a PTC with focal anaplastic changes. The surgical margin was negative. Lymph node metastases were detected in the central, left lateral, left lateral retropharyngeal, and superior mediastinal regions. Consequently, the patient was diagnosed with ATC (pT4aN1bM0, stage IVB). Following the histopathological diagnosis, positron emission tomography/computed tomography was performed two months post-operatively, and early recurrence in the left posterior neck was detected. Considering the properties of ATC and the location of the recurrence, the new lesion was determined to be unresectable. Therapeutic modalities such as chemotherapy, radiotherapy, chemoradiotherapy, and tyrosine kinase inhibitors were considered. Finally, lenvatinib therapy was selected with the expectation of prolonging survival and maintaining good flap condition. Three months post-operatively, daily administration of 24 mg of lenvatinib was started.

**Figure 4:**
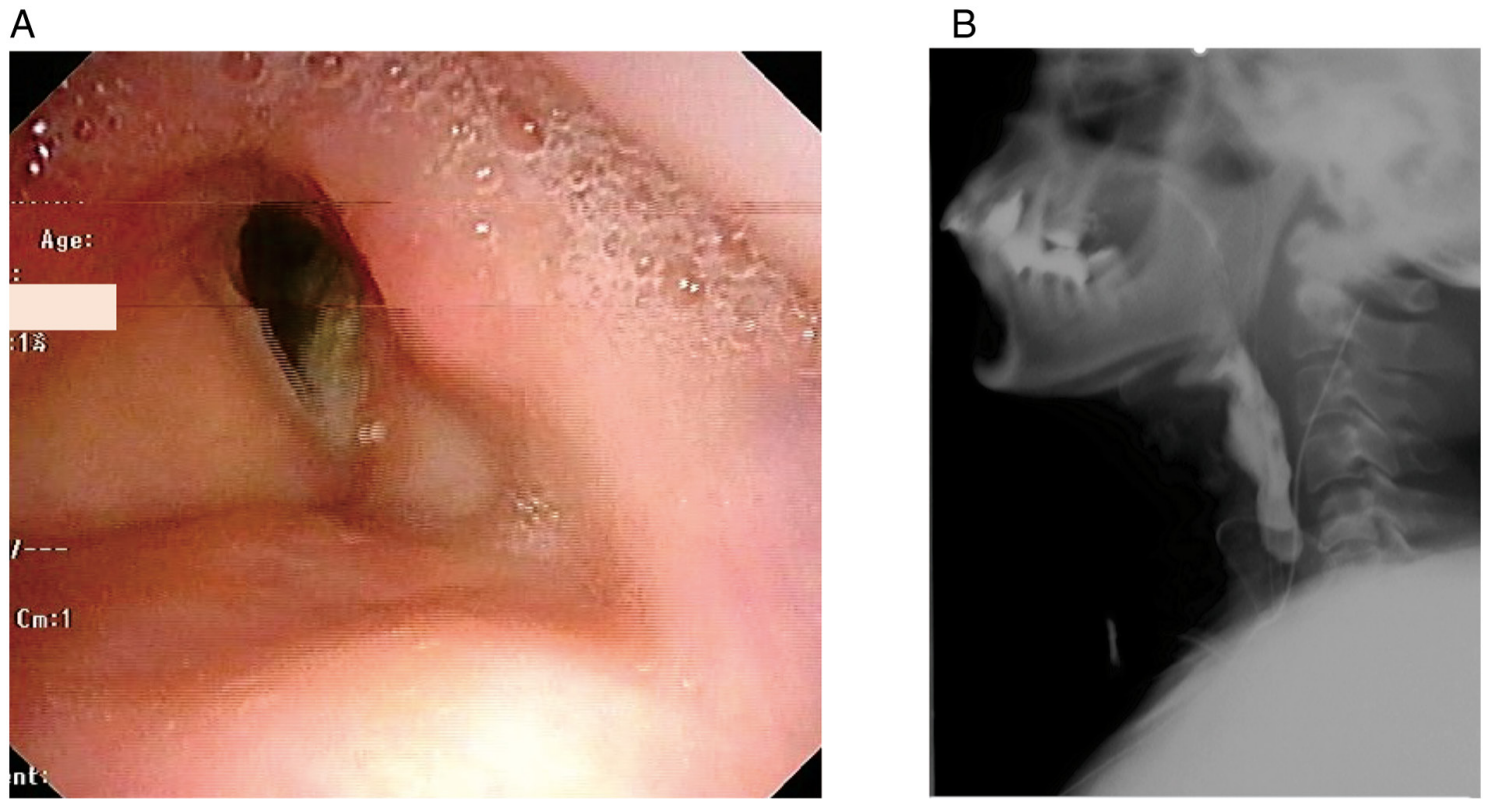
Post-operative findings. (A) Post-operative laryngoscopic findings. The reconstructed larynx had a sufficiently patent lumen. (B) Post-operative videofluorographic image indicated functional swallowing with no leakage.

Twenty-six months after surgery, the patient's good condition had been maintained without any noteworthy adverse effects due to lenvatinib. Positron emission tomography/computed tomography was performed, and we detected no sign of the tumour in the left posterior neck. Post-operative lenvatinib therapy was highly effective and her speech and swallowing functions were approximately the same as those pre-operatively (Fig. 4).

## Discussion

The larynx not only has a complex anatomy but is also involved in multiple functions, such as breathing, speech, and swallowing. It may be directly invaded by carcinomas of the oesophagus or thyroid. It is said that 1-7% of thyroid carcinomas invade the trachea and/or larynx. Advanced thyroid carcinoma occasionally causes tracheal obstruction, leading to death (1,2). Surgical management is thought to be effective in resectable advanced thyroid carcinoma (7,8). However, it involves partial laryngotracheal resection. Our patient's tumour extended beyond the thyroid capsule, that is, from the left wing of the thyroid cartilage to the cricoid cartilage. Therefore, the laryngeal defect was reconstructed after total thyroidectomy and hemi-laryngectomy. Many authors have described the technique of laryngotracheal reconstruction (3,9-12). We used an ALT flap and rib cartilage grafts. An ALT flap is often used as material for head and neck reconstruction. However, there are few reports on its application in laryngeal reconstruction. An ALT flap has adequate size and volume to cover laryngeal defects. In our patient, we were able to use it to sufficiently compensate for a 40% defect of the larynx. It is also easy to harvest in the supine position. Moreover, the post-operative scar at the donor site is hidden behind the patient's clothes, making it relatively inconspicuous in daily life. However, follow-up of the patient's thigh condition is important. Post-operatively, older patients should undergo early rehabilitation to maintain independence in their activities of daily living.

Various materials such as the clavicle, rib cartilage, auricular cartilage, and nasal septum cartilage are used to reinforce the laryngeal lumen in case of reconstruction. We used rib cartilage because it is rigid and easy to harvest and mould. The donor site scar can be easily hidden behind the patient's clothes, similarly to that of an ALT flap. We performed laryngeal reconstruction to maintain airway patency. We vertically inserted the rib cartilage into a pocket immediately below the dermis of the skin flap. Kubo reported the effectiveness of this procedure using a forearm flap (13). As an ALT flap is soft, it is easy to mould it to the irregularly shaped larynx and place trimmed cartilage grafts inside it. Additionally, placement of the graft immediately below the dermis makes it adhere easily and helps to maintain the shape of the reconstructed larynx and trachea. Neck flexibility is also important. Movement of the cartilage pieces makes the neck more flexible. On the other hand, prevention of necrosis or leakage is important.

Although numerous techniques of laryngeal reconstruction have been reported, most of them involve stepwise procedures and palliative stenting. A one-stage procedure could reduce patient burden and help to avoid multiple surgeries. This procedure may lead to early healing of the surgical wound, which may improve patients' quality of life. Post-operative management, including monitoring for signs of infection and inflammation, is important. In our patient, post-operative computed tomography and laryngoscopy showed a reconstructed larynx with sufficient function. Twenty-six months post-operatively, the patient maintained an excellent airway and her larynx functioned adequately.

ATC is a fatal disease for which various therapeutic modalities have been unsuccessfully tried. Moreover, it has been reported that 20-50% of patients with ATC have distant metastasis at initial presentation, and the recurrence rate after treatment is nearly 40% (14,15). This worsens the prognosis. Sugitani *et al* (16,17) devised a prognostic index for ATC based on four unfavourable prognostic factors: Acute symptoms (duration of severe symptoms such as dysphonia, dysphagia, dyspnoea, and rapid tumour growth in <1 month), leukocytosis (leukocyte count 10,000/mm^3^), tumour size >5 cm, and distant metastasis. They recommended attempting multimodal treatment for patients with a prognostic index of ≤1. Our patient did not have any unfavourable prognostic factors.

Our patient experienced recurrence after laryngeal reconstruction using an ALT flap and rib cartilage grafts. While treating the recurrence, we had to focus on the condition of these flaps, which needed sufficient blood supply. Radiotherapy often damages tissues and induces inflammatory responses, such as arteritis and thrombosis in small vessels (18-20). Gessert *et al* (21) reported that the incidence of laryngeal chondroradionecrosis in patients with laryngeal cancer treated with radiotherapy is 2.4%. It is necessary to avoid these complications, maintain the flaps in good condition, and assume that chondroradionecrosis may occur following radiotherapy to the neck (22,23). Radiotherapy can also inhibit post-operative wound healing.

Considering these problems and the post-operative histopathological diagnosis of ATC, a multidisciplinary team consisting of medical oncologists, radiologists, and head and neck surgeons decided to avoid post-operative radiotherapy and to treat the patient with systematic administration of lenvatinib rather than local therapy. This decision led to good outcomes with respect to preservation of laryngeal function and improvement of survival.

Her condition has been maintained at 26 months after the initial treatment. She has good laryngeal function, and we will continue to regularly follow her progress.

In conclusion, one-stage laryngeal reconstruction is highly useful as a treatment for advanced thyroid carcinoma invading the larynx. This procedure could be effective in reducing patient burden and improving quality of life. The use of an ALT flap with free rib cartilage grafts resulted in maintained laryngeal function and neck flexibility. Lenvatinib may be an option for the treatment of early recurrence of ATC after reconstructive surgery with a free flap.

## Data Availability

The datasets used and/or analysed during the current study are available from the corresponding author on reasonable request.
